# A Biocompatible and High-Sensitivity Lanthanide Coordination Complex for Luminescent Temperature Sensor Constructed from Non-Steroidal Anti-Inflammatory Drug Ketoprofen

**DOI:** 10.1021/acsomega.5c11396

**Published:** 2026-02-12

**Authors:** Júlia Pereira de Oliveira Silva, Molíria V. dos Santos, Roberta S. Pugina, Marina Paiva Abuçafy, Francisco R. Torres, José Maurício A. Caiut, Lippy F. Marques

**Affiliations:** † Grupo de Química de Coordenação e Espectroscopia de Lantanídeos (GQCEL), Instituto de Química, 28130 Universidade do Estado do Rio de Janeiro, Rio de Janeiro, RJ 20550-013, Brazil; ‡ Instituto Federal de Educação, Ciência e Tecnologia do Paraná (IFPR), Pitanga, PR 85201-106, Brazil; § BioSmart Nanotechnology, Faculdade de Engenharia Química, Universidade Estadual de Campinas—UNICAMP, Campinas, SP 14808-162, Brazil; ∥ Instituto de Química, Universidade Estadual Júlio de Mesquita Filho—UNESP, Araraquara, SP 14800-060, Brazil; ⊥ Departamento de Química, Grupo de Nanomateriais e Sistemas Luminescentes, Faculdade de Filosofia, Ciências e Letras de Ribeirão Preto, Universidade de São Paulo (USP), Ribeirão Preto, SP 14040-901, Brazil

## Abstract

Temperature is a crucial thermodynamic parameter for sustaining life, driving industrial development, and regulating environmental processes. In this context, luminescence thermometry has emerged as an effective, rapid, and noninvasive method for temperature measurement. In this work, we synthesized a new complex containing Eu^3+^ and Tb^3+^ ions coordinated by 2,2′-bipyridyl (bpy) and the nonsteroidal anti-inflammatory drug ketoprofen (keto), with the empirical formula [Eu_0.4_Tb_1.6_(keto)_6_(bpy)_2_]. The ligand’s T_1_ energy of 23364.48 cm^–1^ makes it effective for sensitizing lanthanide ions. The complex was fully characterized by infrared spectroscopy, elemental (CHN) analysis, powder X-ray diffraction, and thermal analysis. Photophysical studies revealed temperature-dependent emission, with the emission color shifting from yellow at cryogenic temperatures to red at 393 K. This behavior is consistent with a temperature-activated Tb^3+^ → Eu^3+^ energy-transfer contribution, as supported by the decrease in τ­(^5^D_4_) from 1.278 to 0.789 ms between 283 and 343 K. The compound was evaluated as a luminescent thermometer using the intensity ratio between the ^5^D_4_ → ^7^F_5_ (Tb^3+^) and ^5^D_0_ → ^7^F_2_ (Eu^3+^) transitions as the thermometric parameter. The complex exhibited a maximum relative thermal sensitivity of 4.72% K^–1^ at 343 K, a minimum temperature uncertainty of 0.07 K, and reproducibility of 91.7% over the range 283–343 K. Its high sensitivity, together with favorable cell-viability results, indicates that [Eu_0.4_Tb_1.6_(keto)_6_(bpy)_2_] is a promising candidate for use as an efficient luminescent thermometer, including potential applications as a temperature sensor in biological systems.

## Graphical abstract

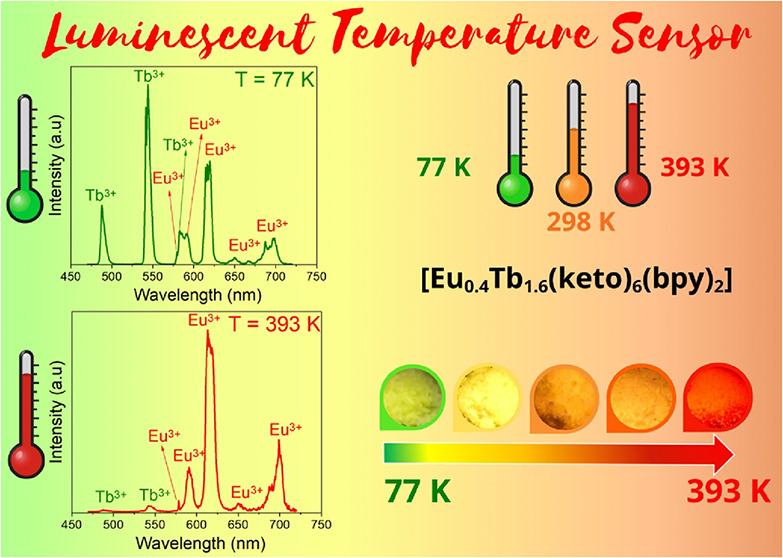

## 1 Introduction

Temperature is a thermodynamic parameter of great importance in a wide range of fields,^1,2^ especially in synthesis processes and the development of new materials, as well as in everyday physical, chemical, and biological phenomena.^3^ Multiple thermometers, with different measuring mechanisms, are commercially available. On the other hand, as highlighted by Dramicanin, luminescent thermometers have been gaining prominence in areas such as nanotechnology, biomedicine, and optoelectronics, where conventional methods have limitations.^4^ Among the luminescent devices, those based on lanthanide ions (Ln^3+^) offer significant advantages, including contactless temperature measurement, high sensitivity, rapid response, and good resolution.^5^ The applicability of these complexes in thermometry arises from their alterations in spectroscopic properties with temperature variation. Spectroscopic parameters such as (i) the integrated intensities of the intraconfigurational 4f-4f transitions of the Ln^3+^; (ii) the displacement of the bands; and (iii) the lifetimes of the emission states of the Ln^3+^ ions,^6^ are commonly monitored to evaluate the effectiveness of the materials as luminescent thermometers.

From spectroscopic measurements (emission, excitation, and decay curves), fundamental indicators are determined, such as relative sensitivity (*S*_r_ > 1% K^–1^), temperature resolution (δ*T*), and repeatability (*R*).^4,7^ *S*_r_ indicates the percentage variation of the thermometric parameter (Δ) based on the degree of temperature change, and δ*T* represents the smallest detectable temperature variation,^8^ and repeatability reflects the precision of the material in successive heating and cooling cycles under the same experimental conditions.^9^ In this context, coordination compounds have demonstrated interesting spectroscopic responses to temperature, being widely reported as luminescent sensors.^10–14^ The synthesis of coordination compounds containing different ratios of Ln^3+^ ions enables the development of novel structures, due to the wide variety of possible combinations among Ln^3+^ and the broad range of available ligands. Such compounds hold a prominent position in the field of Inorganic Chemistry, especially because of their tunable luminescence, which arises from the optical properties of Ln^3+^. Currently, lanthanide coordination compounds are among the primary materials studied for luminescent thermometry, as their emission properties can vary according to ambient temperature.^15^ The literature reports various types of lanthanide coordination compounds, including discrete complexes, coordination polymers, and metal–organic frameworks (MOFs), as material classes that have been extensively investigated for luminescent thermometry, alongside other systems.

The optical properties of lanthanides, including their high emission intensity and long excited-state lifetimes,^16^ have been extensively explored across various research fields. These ions, with atomic numbers ranging from 58 to 71, belong to the sixth period of the periodic table.^17^ Their spectroscopic characteristics arise from intraconfigurational electronic transitions within the 4f*^n^* sublevel, where the electrons are shielded by the filled 5s^2^ and 5p^6^ orbitals. This shielding reduces the interaction between the 4f electrons and the external chemical environment, such as the ligand’s crystal field, resulting in electronic spectra characterized by sharp bands and with monochromatic character.^18–20^ Among the 14 lanthanides, two of the most used emitters in the visible region are europium (Eu^3+^), which emits red light, and terbium (Tb^3+^), which emits green light. The formation of mixed compounds containing different proportions of these ions enables the generation of “tunable luminescence,” due to energy transfer between the metal centers.^21–23^ To enable the observation of such diverse emission colors from the metallic centers, organic ligands must be coordinated to the Ln^3+^, thereby functioning as “antennas”.^24^ These ligands must be capable of absorbing ultraviolet radiation and transferring the energy via nonradiative processes from their triplet state (T_1_) to the excited states of the Ln^3+^.

In this context, a class of ligands capable of acting in such a manner includes the nonsteroidal anti-inflammatory drugs (NSAIDs). NSAIDs are a widely commercialized class of molecules used for pain relief, fever reduction, and flu treatment, due to their analgesic, anti-inflammatory, and antipyretic properties.^25,26^ Lanthanide ions are classified as hard acids according to Pearson’s principle and, therefore, exhibit high affinity for hard bases, such as the carboxylate groups resulting from NSAID deprotonation, leading to the formation of complexes with enhanced luminescent emission.^27,28^ However, when synthesis is carried out using a single NSAID, water molecules or other solvents may coordinate to the metal centers in order to complete the coordination sphere. As a result, radiative deactivation of the lanthanide emissions may occur due to the vibrational modes of O–H oscillators from coordinated water molecules.^29^ To minimize this effect, bidentate nitrogen-containing ligands of the N–N′ donor type, which also behave as hard bases, can be employed to replace water molecules in the first coordination sphere of Ln^3+^ ions.^30^

In this work, we report the synthesis and characterization of a mixed-metal complex containing Eu^3+^ and Tb^3+^ ions, the nonsteroidal anti-inflammatory drug ketoprofen (keto), and the nitrogenous ligand 2,2′-bipyridyl (bpy). The complex was investigated as a luminescent thermometer because it exhibits a systematic temperature-dependent change in emission color. Thermometric studies were performed, allowing calculation of the relative sensitivity over the range 283–343 K. Additional performance parameters relevant to thermometric applications were also evaluated; the results exceed those reported for comparable systems in the literature. Furthermore, cell-viability assays were carried out to assess the biocompatibility of the mixed complex, supporting its potential use as a luminescent temperature sensor in biomedical applications.

## 2 Experimental Section

### 2.1 Materials and Measurements

The 2-(3-benzoylphenyl)­propanoic acid ligand-Ketoprofen (keto) used for the synthesis was obtained from the Fundação Oswaldo Cruz (FIOCRUZ-RJ) and presented 98% purity. The europium chloride hexahydrate salt and terbium chloride hexahydrate salt were used as obtained, that is, without further purification. All lanthanide salts were purchased from Sigma-Aldrich Brazil, as was the nitrogen ligand 2,2′-Bipyridyl (bpy). Inductively Coupled Plasma Spectroscopy (ICP) was used to determine the Eu^3+^/Tb^3+^ ratios in the mixed compound. Samples were prepared by digestion in concentrated HCl (37% w/w), followed by dilution to a 0.5% HCl solution. All Fourier transform near-infrared (FTIR) spectroscopy measurements were performed in ATR mode, with spectra obtained in the wavenumber range 4000–500 cm^–1^ with a spectral resolution of 4 cm^–1^ using a PerkinElmer FTIR/FIR Frontier spectrometer, serial no. C 105496. Thermogravimetric (TG) analysis was carried out in the temperature range 50–800 °C using approximately 3.5 mg of the compounds under a nitrogen atmosphere with a heating rate of 10 °C/min. These measurements were carried out on a model Q50 equipment from TA Instruments, USA. Powder X-ray diffraction (PXRD) of the obtained complex was performed on a D8 ADVANCE diffractometer with Cu Kα radiation (1.542 Å) with a tube voltage parameter of 45 kV, current of 40 mA, and Bragg–Brentano geometry. Measurements were performed with a 2θ range from 5 to 30 °C and a step angle of 0.02° at room temperature. Photoluminescence spectra at room temperature were obtained using a Jobin-Yvon Fluorolog-322 spectrofluorimeter. The equipment is equipped with a Hamamatsu R928 photomultiplier and a 450 W xenon lamp as the excitation source. The equipment contains a TRIAX 320 dual monochromator for both excitation and emission. However, for the thermometry study, temperature-dependent emission spectra were obtained using a Horiba Fluorolog 3, model FL3–22, with dual excitation and emission monochromators and Hamamatsu R928 photomultipliers. Spectra were collected over the temperature range of 77 to 393 K. The sample was placed under a platinum crucible in a LINKAM Scientific temperature controller (T95-HT). The sample temperature was stabilized before capturing each spectrum. An optical fiber coupled to the Horiba Scientific Fluorolog 3 was responsible for exciting the sample, and another optical fiber collected the emission spectra at each temperature of the experiment. Quantum yield measurement was performed using a Hamamatsu Quantaurus-QY Plus UV-NIR absolute photoluminescence quantum yield spectrometer, equipped with a 150 W xenon lamp as the excitation source. The setup includes a 3.3-in. Spectralon integrating sphere and a CCD detector operating over the 300–950 nm spectral range. The solid sample was mounted in a quartz holder and positioned on a Spectralon base within the integrating sphere for measurement.

### 2.2 Synthesis of the [Eu_0.4_Tb_1.6_(keto)_6_(bpy)_2_] Complex

The syntheses and characterization of the homometallic complexes [Eu_2_(keto)_6_(bpy)_2_] and [Tb_2_(keto)_6_(bpy)_2_] have been reported previously, and some of their data were used here as references.^31^ For the preparation of the mixed complex [Eu_0.4_Tb_1.6_(keto)_6_(bpy)_2_], 0.57 mmol of 2-(3-benzoylphenyl)­propanoic acid (ketoprofen, keto) was placed in a 50 mL beaker containing 10 mL of distilled water. The aqueous suspension was stirred and then deprotonated by the dropwise addition of 0.57 mL of 1.00 M NaOH (0.57 mmol). Immediately thereafter, two solutions were added simultaneously to the reaction mixture. The first solution consisted of 0.19 mmol of lanthanide chloride hexahydrates (EuCl_3_·6H_2_O and TbCl_3_·6H_2_O), dissolved in 10 mL of distilled water, with the molar fractions adjusted to achieve the target composition (20 mol % EuCl_3_·6H_2_O and 80 mol % TbCl_3_·6H_2_O). The second solution comprised 0.19 mmol of 2,2′-bipyridyl (bpy) dissolved in 5 mL of ethanol. Upon simultaneous addition of the lanthanide and ligand solutions to the ketoprofen suspension, a white solid precipitated immediately. The reaction mixture was stirred at room temperature for 24 h. The resulting solid was then collected by filtration, washed several times with distilled water followed by ethanol, and stored in a desiccator. The synthesis scheme is shown in Figure 1. Yield: 74.6% for [Eu_0.4_Tb_1.6_(keto)_6_(bpy)_2_]. Anal. Calc. for [Eu_0.4_Tb_1.6_(keto)_6_(bpy)_2_]: C_116_H_94_O_18_N_4_Eu_0.4_Tb_1.6_: %C: 64.89, %H: 4.41 and %N: 2.61; Found: %C: 65.01, %H: 4.40 and %N: 2.62. (Eu^3+^: Tb^3+^) ICP analysis: %Eu: 0.3899 and %Tb: 1.6019.

**Figure 1:**
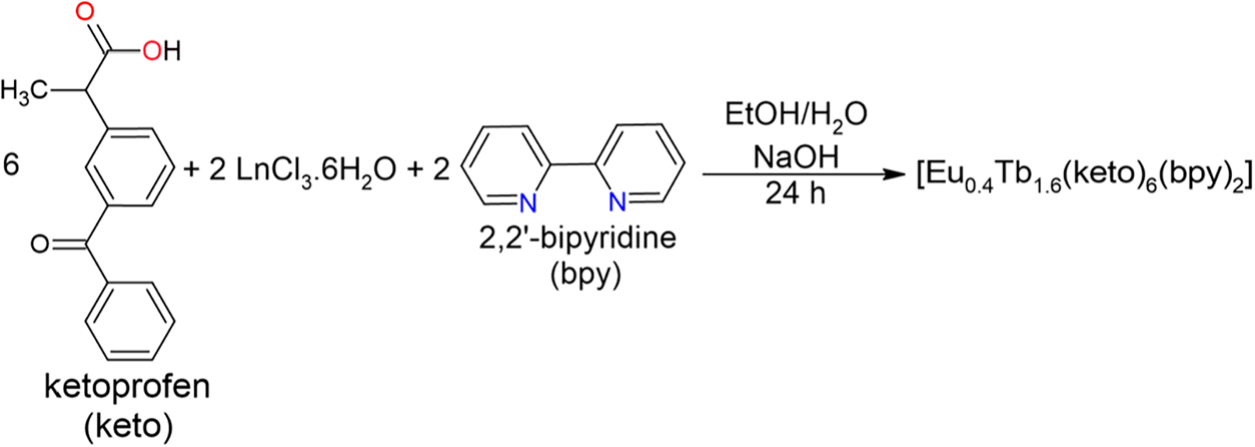
Synthesis scheme of the mixed complex.

### 2.3 Cell Culture and Cellular Viability

The in vitro cytotoxicity assay of the heterometallic complex [Eu_0.4_Tb_0.6_(keto)_6_(bpy)_2_], and its ligands ketoprofen (keto) and 2,2′-bipyridine (bpy) was assessed using the nontumoral human lung fibroblast cell line MRC-5 (ATCC CCL-171). The cells were maintained under standard culture conditions at 37 °C in a humid atmosphere containing 5% CO_2_. Routine cultivation was carried out in Dulbecco’s Modified Eagle Medium (DMEM, high glucose; Gibco, USA) supplemented with 10% (v/v) fetal bovine serum (FBS; Gibco, USA), penicillin (100 U mL^–1^), and streptomycin (100 μg mL^–1^). For cytotoxicity experiments, the cells (1 × 10^4^ cells/100 μL) were seeded into 96-well and kept at 37 °C and 5% CO_2_ for 24 h in a cell culture incubator.^32^ The next day, different concentrations of complexes and its ligands diluted in DMSO (dimethyl sulfoxide; Sigma-Aldrich, USA) (0.39–100 μM) were added to the wells. The cells were incubated for 48 h in the same conditions described above, and after the incubation a new culture media containing 0.5 mg mL^–1^ of MTT ([3-(4,5-dimethylthiozol-2-yl)-2,5-diphenyltetrazolium bromide]; Sigma-Aldrich, USA) was added (50 μL/well) and the plates were incubated for additional 4h at 37 °C. After this time, the crystals formed were diluted in DMSO and the absorbance of the conversion of MTT to formazan by metabolically viable cells was read on microplate reader (Biotek Synergy HTX) at a wavelength of 570 nm, according to Mosmann^33^ The viability of treated cells was normalized to that of negative control cells (without treatment). The complex’ IC_50_ (concentration that induces 50% cell death) was determined from a concentration curve using the (GraphPad Prism software 9.0). Viability data represent *n* = 3 independent experiments and were analyzed by one-way ANOVA.

## 3 Results and Discussion

### 3.1 Vibrational (IR) Spectroscopy, TG Curve, and X-ray Powder Diffraction Studies

In the infrared spectra, the main characteristic bands of the ligands and of the synthesized mixed complex are highlighted in Figure 2. Ketoprofen (keto, Figure 2a) exhibits a broad ν­(O–H) stretching band typical of carboxylic acids, centered at 3450 cm^–1^. Bands corresponding to the aromatic C–H stretching vibrations appear at 2979 cm^–1^, together with ν­(C=C) stretching bands at 1599 and 1457 cm^–1^. Bands associated with out-of-plane C–H deformations (γ­(C–H)) are observed at 810, 770, and (ν­(C–H)) 691 cm^–1^. In addition, ketoprofen shows a band at 1697 cm^–1^, attributed to the C=O stretching vibration of the carboxylic acid, and a band at 1656 cm^–1^ assigned to the ν­(C=O) stretching of the ketone group. After deprotonation, the spectrum of sodium ketoprofenate (Naketo, Figure 2b) shows the disappearance of the broad ν­(O–H) band, confirming the formation of the ionic carboxylate. In this spectrum, the carboxylate anion displays an asymmetric stretching band, ν_asym_(COO^–^), at 1588 cm^–1^ and a symmetric stretching band, ν_sym_(COO^–^), at 1395 cm^–1^, giving [Δν = ν_asym_(COO^–^) – ν_sym_(COO^–^) = 193 cm^–1^]. The infrared spectrum of the nitrogenous ligand (bpy, Figure 2c) displays the characteristic absorptions of an aromatic heterocycle. In the aromatic region, a C–H stretching band appears at 3053 cm^–1^, together with ν­(C=C) bands at 1586 and cm^–1^ and a ν­(C=N) stretching band at 1560 cm^–1^. In the spectrum of the mixed complex [Eu_0.4_Tb_1.6_(keto)_6_(bpy)_2_] (Figure 2d), the characteristic ν_asym_(COO^–^) and ν_sym_(COO^–^) bands are split into two sets, indicating the presence of at least two distinct coordination environments for the carboxylate group.^34^ For this complex, the observed Δν values are approximately 196 cm^–1^ and 112 cm^–1^. The larger value (Δν complex ≈ 196 cm^–1^ ≈ Δν Naketo) is consistent with a bridging coordination mode, whereas the smaller value (Δν complex ≈ 112 cm^–1^ < Δν Naketo) suggests a bidentate chelating coordination mode. Thus, the IR spectra confirm the deprotonation of ketoprofen and its coordination to the lanthanide centers, and they also highlight the contribution of the bipyridyl ligands to the overall vibrational profile of the complexes.

**Figure 2:**
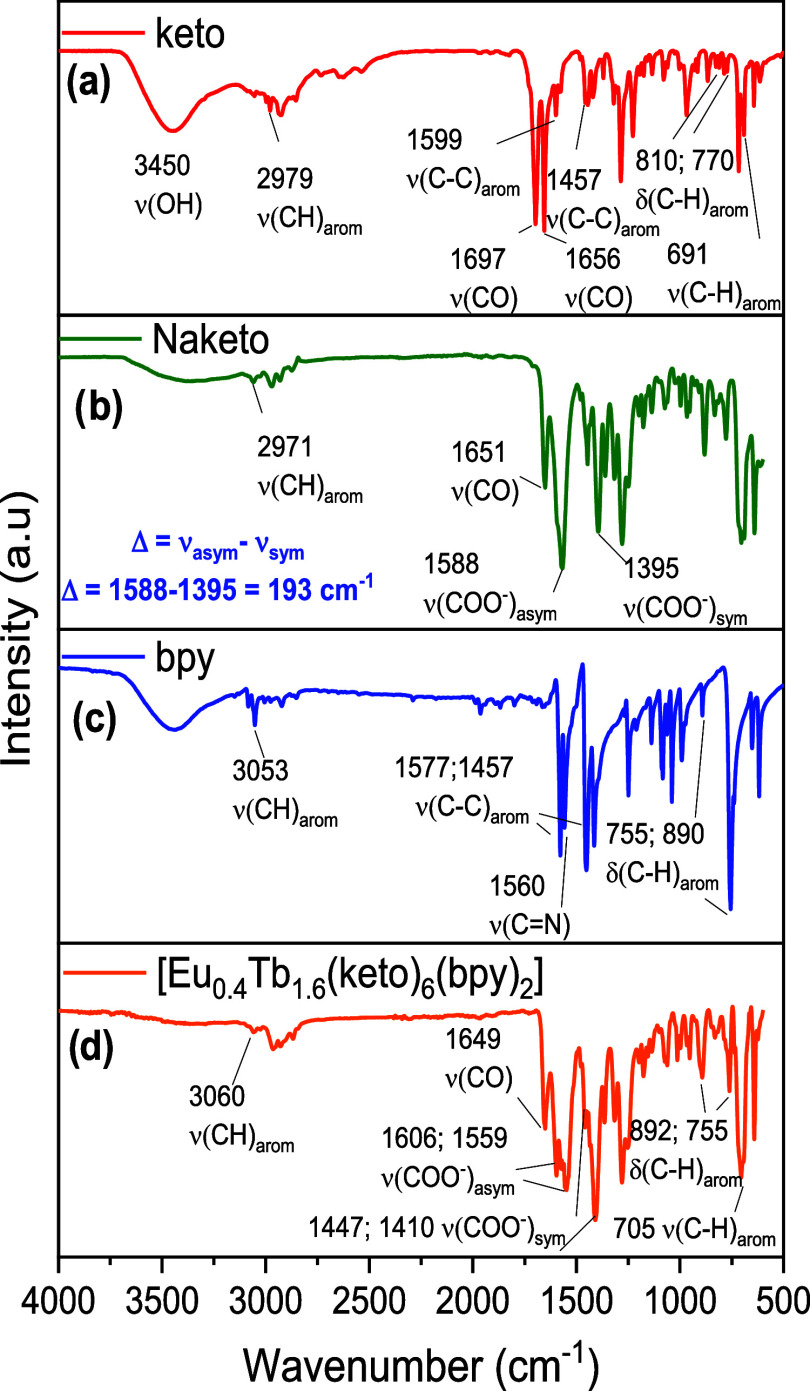
Infrared spectra of (a) ketoprofen (red); (b) sodium salt Naketo (green); (c) nitrogenous ligand (blue), and (d) mixed complex (orange).

The thermogravimetric (TG) data shown in Figure 3 are crucial for evaluating the thermal stability of the mixed complex and for identifying any coordinated or lattice solvent molecules. The complex [Eu_0.4_Tb_1.6_(keto)_6_(bpy)_2_] exhibits no significant mass loss at low temperatures, indicating the absence of volatile solvent molecules (e.g., water or ethanol) in the solid. This observation is consistent with the elemental and vibrational analyses.

**Figure 3:**
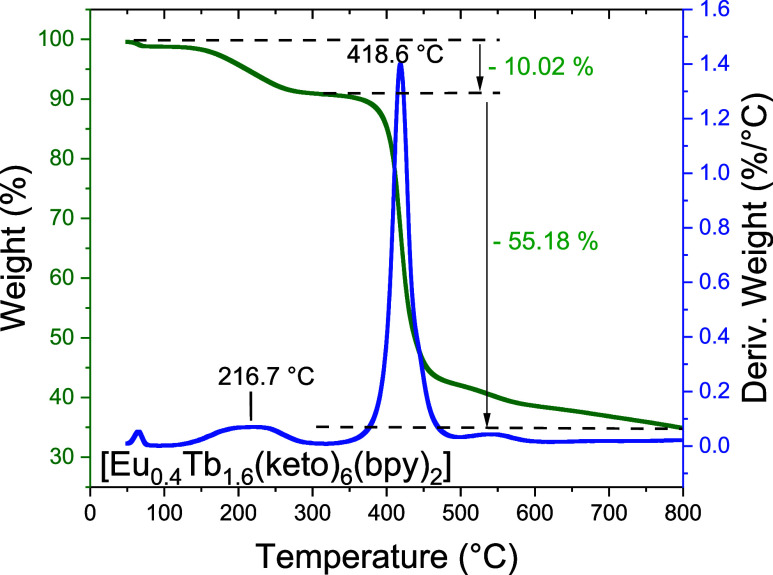
TG (green) and dTG (blue) curves of the [Eu_0.4_Tb_1.6_(keto)_6_(bpy)_2_] complex.

The first mass loss of approximately 10.02% occurs during the initial decomposition stage and can be attributed to the loss of two 2,2′-bipyridyl ligands; the theoretical mass loss for this process is 14.55%. A second, more pronounced mass loss of 55.18% (with a maximum rate at 418.6 °C in the DTG curve) corresponds to the decomposition of the six ketoprofenate ligands (calculated: 57.76%). At 800 °C, a residual mass of approximately 35% is observed, which can be ascribed to the formation of the corresponding metal oxides together with carbonized residues remaining after the complete decomposition of the organic components. The small discrepancies between experimental and calculated mass losses may reflect overlapping decomposition steps, partial volatilization of decomposition products, residual solvents below the detection limit, or experimental uncertainty in the TG measurement.

The X-ray powder diffraction (PXRD) pattern of the mixed complex [Eu_0.4_Tb_1.6_(keto)_6_(bpy)_2_] is similar to the diffraction profiles of the corresponding homometallic complexes [Ln_2_(keto)_6_(bpy)_2_] (Ln = Eu^3+^, Tb^3+^) previously reported by our research group.^31^ As shown in Figure S1, the PXRD pattern of the mixed complex lacks well-defined, sharp Bragg peaks, indicating an amorphous character. However, it is well established in the literature that coordination compounds formed by monocarboxylate anions and *N*,*N*′-bidentate ligands can yield homobimetallic complexes, with some examples of crystalline structures reported.^35–37^ Given the inherent difficulty in obtaining structural data for mixed complexes, we adopted an approach based on homobimetallic analogues,^31^ using spectroscopic and thermal analyses to corroborate the proposed structural similarity. To illustrate this discussion, Figure 4a presents the molecular structure of the heterobimetallic complex based on the homobimetallic complex [Eu_2_(keto)_6_(bpy)_2_].^31^ The strong similarity observed among the thermogravimetric curves, infrared spectra, and X-ray powder diffraction patterns of the homobimetallic compounds and the mixed complex [Eu_0.4_Tb_1.6_(keto)_6_(bpy)_2_] strongly suggests that this system is isostructural. In particular, the thermal decomposition profiles and the vibrational modes assigned to the carboxylate groups are fully consistent with the coordination modes identified in the homobimetallic analogues. Figure 4b,4c,4d depict different coordination modes of the COO^–^ anions to the metal centers: in (b), ketoprofenate adopts a *syn,syn*-η^1^:η^1^:μ_2_ coordination mode; in (c), a *syn,syn*-η^1^:η^1^ mode is observed; and in (d), the carboxylate ligand coordinates in an η^1^:η^1^:μ_2_ fashion. These findings are in full agreement with the results obtained from vibrational spectroscopy.

**Figure 4:**
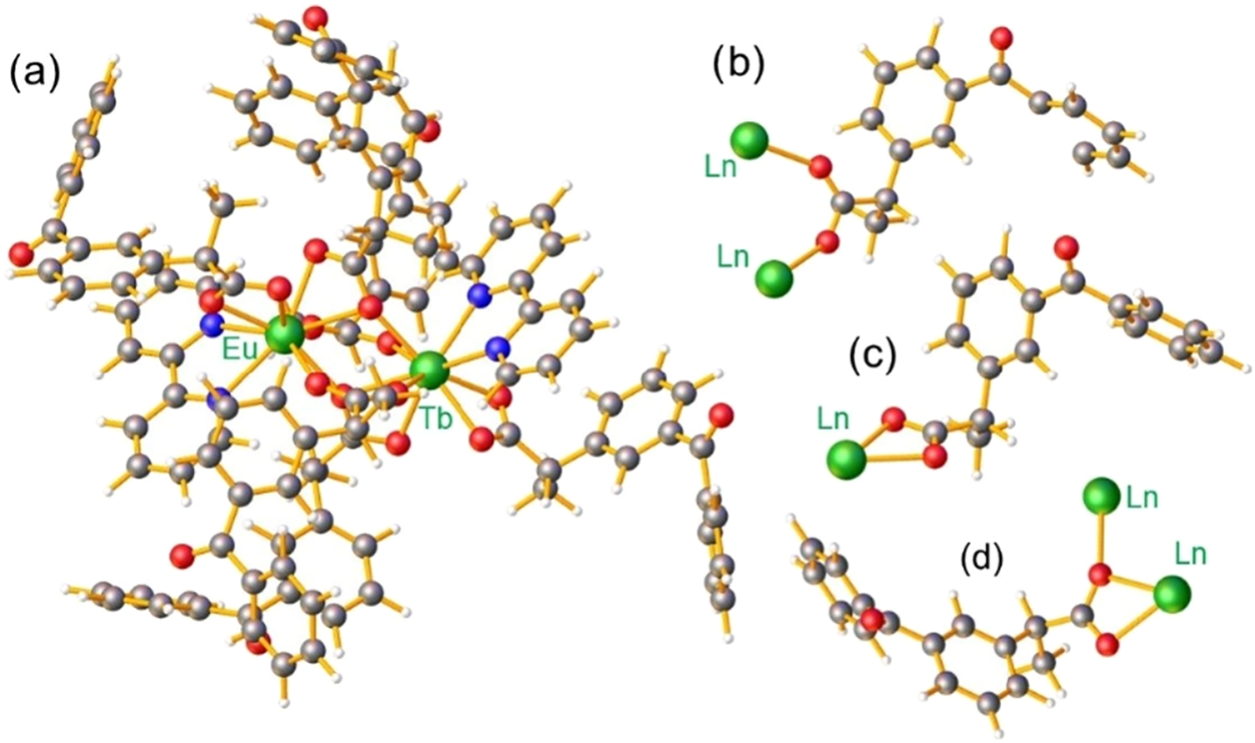
(a) Molecular structure of the heterobimetallic [Eu_0.4_Tb_1.6_(keto)_6_(bpy)_2_] complex; (b–d) coordination modes of the carboxylate groups. The colors of the atoms are represented by gray—carbon; light blue—hydrogen; red—oxygen; dark blue—nitrogen and green—europium.

### 3.2 Photoluminescence Study

The emission spectrum of the [Gd_2_(keto)_6_(bpy)_2_] complex was used to determine the energy of the ligand triplet state (T_1_).^31^ The lowest excited state of the Gd^3+^ ion (^6^P_7/2_ ≈ 36,900 cm^–1^) lies at higher energy than the ligand triplet, and therefore energy transfer from the ligands to Gd^3+^ is not expected.^38–41^ For [Gd_2_(keto)_6_(bpy)_2_], the T_1_ energy was estimated from the 0–0 transition at 428.95 nm (23,364.48 cm^–1^). This value indicates that the T_1_ state can sensitize Eu^3+^ and Tb^3+^, since it lies above the emissive levels of Eu^3+^ (^5^D_1_ ≈ 19,027 cm^–1^; ^5^D_0_ ≈ 17,293 cm^–1^) and Tb^3+^ (^5^D_4_ ≈ 20,566 cm^–1^).^42–46^ Thus, the chosen ligands are suitable for efficient sensitization of Eu^3+^ and Tb^3+^ in the mixed complex [Eu_0.4_Tb_1.6_(keto)_6_(bpy)_2_], as confirmed by the spectroscopic evidence.^47–49^

The excitation spectrum of the mixed complex [Eu_0.4_Tb_1.6_(keto)_6_(bpy)_2_] was recorded in the solid state between 250 and 500 nm, monitoring both the Eu^3+^ emission at 617 nm (^5^D_0_ → ^7^F_2_) and the Tb^3+^ emission at 547.5 nm (^5^D_4_ → ^7^F_5_) at 300 K (see Figure S2). The spectra show a broad absorption band from 250 to 400 nm, attributed to the ligand π → π* transition (S_0_ → S_1_). A closer inspection of the enlarged spectra reveals characteristic intraconfigurational 4f–4f transitions: the ^7^F_0_ → ^5^D_2_ transition of Eu^3+^ (≈21,505 cm^–1^) and the ^7^F_6_ → ^5^D_4_ transition of Tb^3+^ (≈20,492 cm^–1^) were observed.

The emission spectrum of [Eu_0.4_Tb_1.6_(keto)_6_(bpy)_2_] (Figure 5a) was recorded at 300 K over the range 465–750 nm. Upon excitation at 330 nm, within the ligand S_0_ → S_1_ band (see Figure S2), intersystem crossing (S_1_ → T_1_) occurs, followed by ligand → metal energy transfer from the ligand triplet state (T_1_) to the ^5^D_4_ level of Tb^3+^ and subsequently to its ^7^F*_n_* manifold. For Tb^3+^, the characteristic intraconfigurational 4f–4f transitions ^5^D_4_ → ^7^F_6_, ^7^F_5_, and ^7^F_4_ are observed at 491, 547.5, and 586.5 nm, respectively. For Eu^3+^, excitation of the ligand system most likely populates the ^5^D_2_ level, which is in better resonance with the T_1_ state; internal conversion then populates the lower ^5^D_1_ and ^5^D_0_ states. The ensuing intraconfigurational transitions ^5^D_0_ → ^7^F_0–4_ appear at 583, 595, 617, 652.5, and 701 nm, respectively.^50,51^ This ligand → metal energy transfer scheme for [Eu_0.4_Tb_1.6_(keto)_6_(bpy)_2_] is summarized in Figure 6. Under these conditions, the ^5^D_4_ → ^7^F_5_ transition of Tb^3+^ and the ^5^D_0_ → ^7^F_2_ transition of Eu^3+^ are the most intense, accounting for the characteristic green and red emissions of the respective ions. Figure 6 presents an inset with a tube containing [Eu_0.4_Tb_1.6_(keto)_6_(bpy)_2_] under 365 nm UV-flashlight excitation, highlighting its strong yellow–orange luminescence. The ^5^D_4_ → ^7^F_5_ transition shows the higher relative intensity, yielding an overall orange–yellow emission color, as illustrated in the Commission Internationale de l’Éclairage (CIE) chromaticity diagram (Figure 5b). The mixed complex [Eu_0.4_Tb_1.6_(keto)_6_(bpy)_2_] exhibited a high photoluminescence quantum yield (Φ) of 95.2%, which confirms its excellent light-emitting properties. To verify the formation of a true mixed complex rather than a simple physical mixture of the homometallic species, the comparative spectra displayed in Figure S3 were recorded. Figure S3 compares the emission spectrum of the mixed complex with that of a physical mixture (20:80 by weight) of the homobimetallic complexes [Eu_2_(keto)_6_(bpy)_2_] and [Tb_2_(keto)_6_(bpy)_2_]. In the physical mixture (green trace), the ^5^D_4_ → ^7^F_5_ transition of Tb^3+^ dominates over the ^7^F_0_ → ^5^D_2_ transition of Eu^3+^, reflecting the higher proportion of Tb^3+^. In contrast, for the mixed complex (red trace), the ^7^F_0_ → ^5^D_2_ transition of Eu^3+^ is nearly as intense as the ^5^D_4_ → ^7^F_5_ transition of Tb^3+^, consistent with efficient Tb^3+^ → Eu^3+^ energy transfer. This distinction is also evident in the CIE diagram, where the physical mixture displays a green emission while the mixed complex exhibits an orange emission.

**Figure 5:**
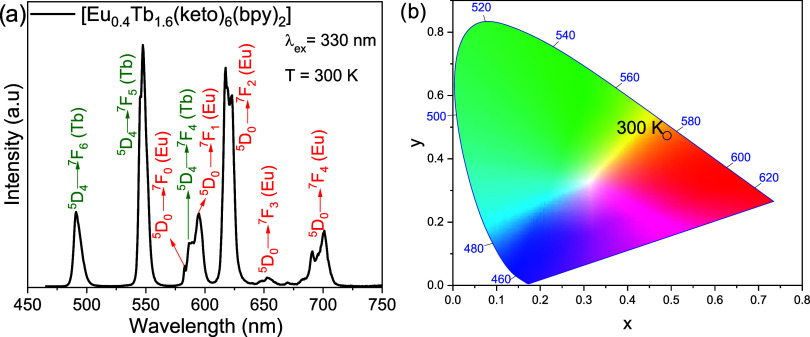
(a) Emission spectrum of the [Eu_0.4_Tb_1.6_(keto)_6_(bpy)_2_] complex, excited at 330 nm in the solid state and recorded at 300 K. (b) CIE chromaticity diagram showing the *x*, *y* (*x* = 0.49026, *y* = 0.47318) emission color coordinates of [Eu_0.4_Tb_1.6_(keto)_6_(bpy)_2_] complex at 300 K.

**Figure 6:**
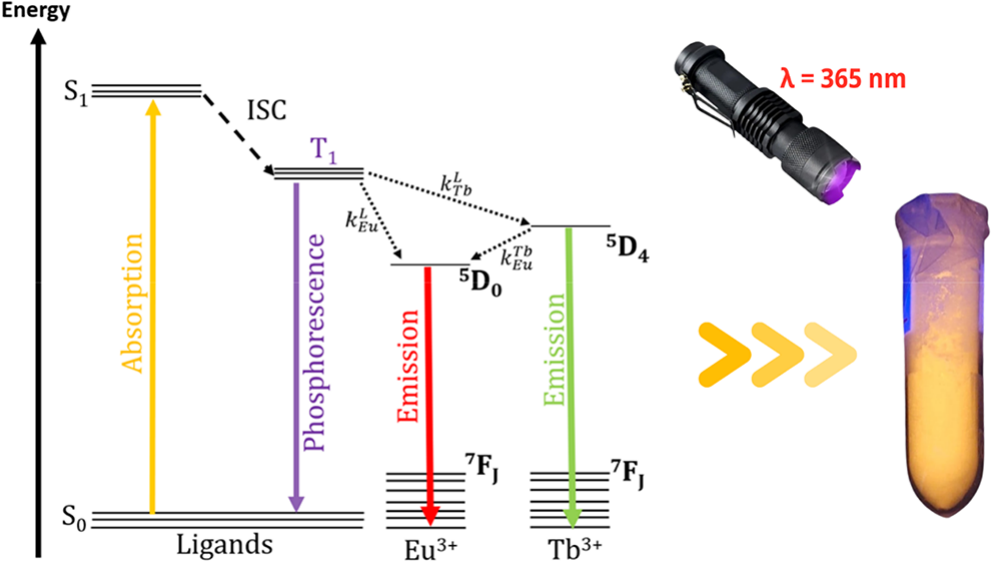
Simplified schematic representation of the energy transfer diagram of the complexes. The solid orange arrow represents the S_0_ → S_1_ ligand absorption (where S = singlet, T = triplet, ISC = intersystem crossing, and *k* = the radiative or nonradiative transition probability). The dotted arrows indicate nonradiative processes, while the straight downward arrows correspond to luminescence from the Eu^3+^ center (red) and from the Tb^3+^ center (green). On the right, the intense emission of [Eu_0.4_Tb_1.6_(keto)_6_(bpy)_2_] is observed when excited by a portable UV flashlight λ = 365 nm.

### 3.3 Temperature-Dependent Luminescence Study for [Eu_0.4_Tb_1.6_(keto)_6_(bpy)_2_]

To evaluate the potential of the complex for luminescent thermometry, temperature-dependent emission spectra were collected over the range 77–393 K upon the excitation at 330 nm of the ligand absorption band. Spectra were recorded at 10 K intervals between 93 and 293 K and between 373 and 393 K, and at 5 K intervals between 273 and 353 K. Figure 7 shows the emission profiles of the Eu^3+^ and Tb^3+^ ions as a function of temperature. Each spectrum was acquired 5 min after the system reached the target temperature to ensure thermal equilibration of the sample. In these spectra the intensity of the ^5^D_4_ → ^7^F_5_ transition (Tb^3+^) decreases with increasing temperature, while the ^5^D_0_ → ^7^F_2_ transition (Eu^3+^) increases in intensity. To examine the different energy-transfer pathways as a function of temperature, emission spectra obtained under direct excitation of the Tb^3+^ ion (at 488 nm) were recorded (Figure S4 in the Supporting Information). These spectra exhibit the same behavior observed when the complex is excited through the ligand system (λ = 330 nm), namely, an increase in the Eu^3+^ emission and a decrease in the Tb^3+^ emission with increasing temperature, suggesting a Tb^3+^ → Eu^3+^ energy-transfer pathway.^52^ Figure S5a displays the same emission spectra as Figure 7 in a 3D projection, with temperature plotted on one axis, which allows clearer visualization of the variation in luminescence intensity as a function of temperature. Figure S5b presents the CIE chromaticity diagram, where the temperature-dependent change in emission color is evident: the chromaticity coordinates shift from (*x* = 0.44817, *y* = 0.50480) at 77 K to (*x* = 0.65077, *y* = 0.34680) at 393 K (see Table S1), reflecting a change from greenish-yellow emission at cryogenic temperatures to reddish emission at higher temperatures. Figure S5c compares the temperature-dependent normalized emission intensities of the ^5^D_4_ → ^7^F_5_ (Tb^3+^) and ^5^D_0_ → ^7^F_2_ (Eu^3+^) transitions.

**Figure 7:**
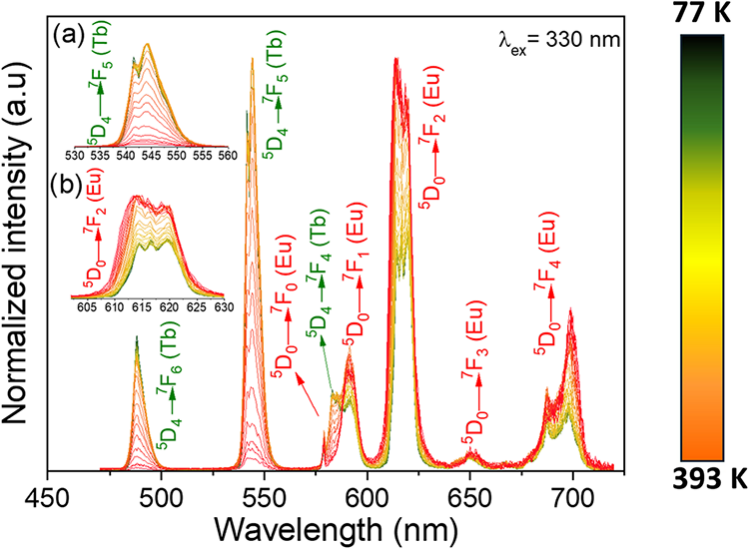
Emission spectra of the [Eu_0.4_Tb_1.6_(keto)_6_(bpy)_2_] complex were recorded at various temperatures ranging from 77 to 393 K, under excitation at the ligand absorption band (330 nm). The spectra were measured in 10 K increments from 93 to 273 K and from 373 to 393, and in 5 K increments from 273 to 353 K. The inset (a, b) highlights the temperature-dependent variations in the Tb^3+^ and Eu^3+^ electronic transition intensities.

Thermometric measurements were performed over the range 283–343 K. In addition, a cell-viability assay was conducted to evaluate the material’s cytotoxicity. Figure 8 presents the thermometric (temperature-dependent luminescence) characterization of the mixed complex [Eu_0.4_Tb_1.6_(keto)_6_(bpy)_2_], obtained under excitation at 330 nm (the ligand absorption band) across the 283–343 K interval. The thermometric parameter chosen to evaluate the complex as a potential temperature sensor was the ratio between the integrated emission areas of the Tb^3+^ (^5^D_4_ → ^7^F_5_) and Eu^3+^ (^5^D_0_ → ^7^F_2_) bands, calculated from the emission spectra, as widely reported for lanthanide-based systems.^53–57^ The thermometric parameter (Δ) was defined as Δ = *A*_Tb_(^5^D_4_ → ^7^F_5_)/*A*_Eu_(^5^D_0_ → ^7^F_2_),^58^ where *A*_Tb_ and *A*_Eu_ are the integrated emission areas for Tb^3+^ and Eu^3+^, respectively.^58^ Integration was performed over 530–560 nm for Tb^3+^ and 602–632 nm for Eu^3+^. Figure 8a presents three-dimensional emission spectra of the complex as a function of temperature (283–343 K), showing a gradual change in the relative intensities of the emission bands, particularly those of Tb^3+^. Figure 8b shows the CIE chromaticity diagram, which documents the shift in emission color from the orange–yellow region at lower temperatures to the red region at higher temperatures. This behavior is attributable to efficient Tb^3+^ → Eu^3+^ energy transfer: the ^5^D_0_ → ^7^F_2_ transition of Eu^3+^ becomes increasingly dominant relative to the ^5^D_4_ → ^7^F_5_ transition of Tb^3+^ (see Figure 8c). In Figure 8c, the emission intensity of Tb^3+^ decreases gradually with increasing temperature, whereas the emission intensity of Eu^3+^ tends to increase.^59,60^ As illustrated in Figure 8d, the parameter Δ is temperature-dependent and is well described by a linear fit (*R* ^2^ = 0.9908), according to (eq 1).

1 Δ=−0.0144T+5.21

**Figure 8:**
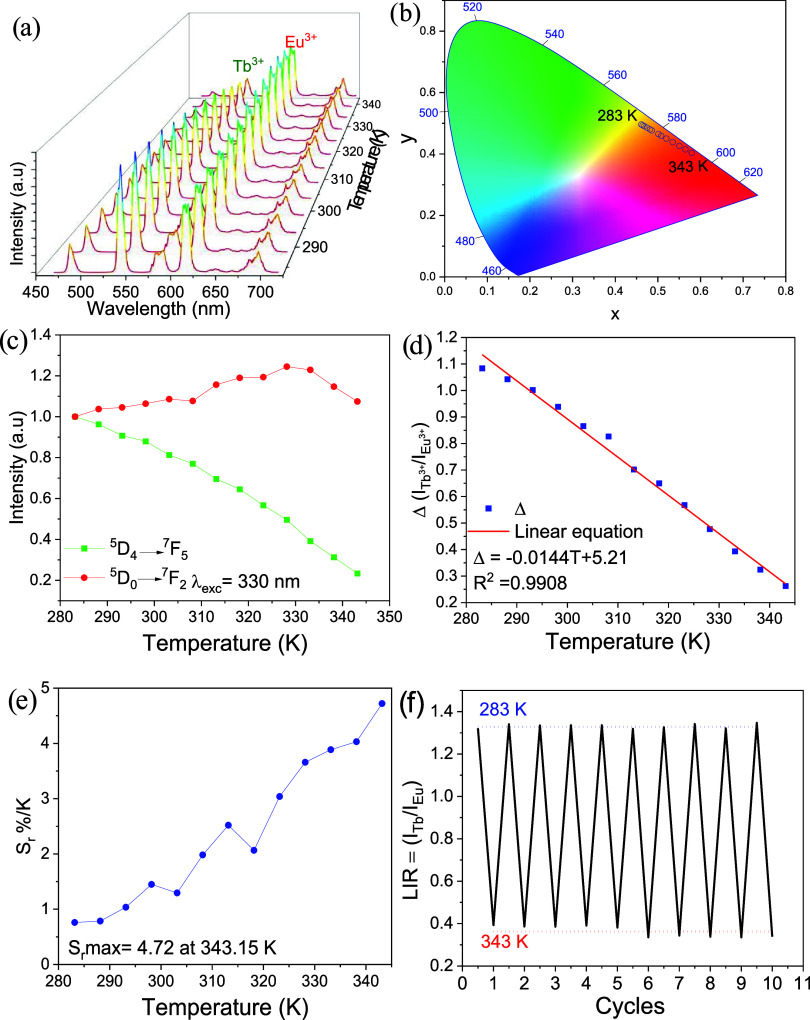
(a) Emission spectra of [Eu_0.4_Tb_1.6_(keto)_6_(bpy)_2_] complex recorded from 283 to 343 K with 5 K step under excitation at the ligand absorption band (330 nm); (b) CIE 1931 chromaticity diagram showing the temperature-dependent color change from greenish-yellow at 283 K to red at 343 K; (c) normalized temperature-dependent intensities of the ^5^D_4_ → ^7^F_5_ and ^5^D_0_ → ^7^F_2_ transitions in [Eu_0.4_Tb_1.6_(keto)_6_(bpy)_2_] collected in the 283 to 343 K range; (d) temperature-dependent intensity ratio of Tb^3+^ (547.5 nm) to Eu^3+^ (617 nm), including the fitted curve for [Eu_0.4_Tb_1.6_(keto)_6_(bpy)_2_] (e) plot showing the relative sensitivity *S*_r_ values at different temperatures (283–343 K); (f) repeatability of the luminescence intensity ratio (LIR) between Tb^3+^ (547.5 nm) and Eu^3+^ (617 nm) under temperature cycling between 283 and 343 K for ten consecutive cycles.

The relative sensibility (*S*_r_) is a fundamental parameter used to evaluate the capacity of a material as a temperature sensor^61^ (eq 2).

2 $$S_{r}=\frac{1}{\Delta }\cdot \left|\frac{\partial \Delta }{\partial T}\right|\cdot 100\%$$

The relative thermal sensitivity *S*_r_ of the [Eu_0.4_Tb_1.6_(keto)_6_(bpy)_2_] complex is shown in Figure 8e; the maximum value observed is 4.72% K^–1^ at 343 K. This sensitivity exceeds those of several comparable mixed complexes reported in the literature (Table 1), indicating that [Eu_0.4_Tb_1.6_(keto)_6_(bpy)_2_] is a highly promising candidate for applications in solid-state luminescent thermometry. Repeatability (*R*) evaluates the thermal stability of the compound under repeated measurements performed under identical conditions.^62^ In other words, repeatability measures the ability of the luminescent thermometer to reproduce consistent results across multiple measurement cycles. This parameter is calculated as given in (eq 3).

3 $$R\;\left(\%\right)=\left[1-\frac{\max\left(|\Delta_{m}-\Delta_{i}|\right)}{\Delta_{m}}\right]\cdot 100\%$$

Here, Δ*_m_* denotes the mean value of the thermometric parameter for the two temperatures in each cycle, and Δ*_i_* is the value of the thermometric parameter for each specific measurement.^63^ A common methodology for evaluating repeatability consists of performing the same measurement ten times and determining the luminescence intensity ratio (LIR) of the emitting centers at the minimum and maximum operating temperatures.^64^ Figure 8f presents the repeatability results obtained over ten measurement cycles for the [Eu_0.4_Tb_1.6_(keto)_6_(bpy)_2_] complex, with temperature cycled between 283 and 343 K. The data show excellent repeatability within this range, with *R* = 98.8% at 283 K and *R* = 91.7% at 343 K (see eq 3). These values indicate that the maximum experimental deviation observed during the heating–cooling cycles is below 9%.^64^ Temperature uncertainty (δ*T*), also referred to as temperature resolution, quantifies the smallest temperature change that produces a detectable variation in the thermometric parameter Δ.^61^ This parameter is calculated using (eq 4)­

4 $$\delta T=\frac{1}{S_{r}}\frac{\delta \Delta }{\Delta }$$

where δΔ/Δ represents the relative uncertainty, calculated based on the relative standard deviation of all the measurements of the thermometric parameter. The mixed complex exhibited a temperature uncertainty in the range of 0.075 to 0.449 K (see Figure S6), a performance considered excellent, given that the reference range for high-precision materials varies from 0.01 to 1 K.^64^

**Table 1:** Comparative Analysis of the Relative Temperature Sensitivity (*S*_r_) of Various Ratiometric Lanthanide-Based Systems Reported in the Literature^a^

complexes	range (K)	*S*_r_ (max) %K^–1^	references
[Tb_0.99_Eu_0.01_TCT]	303–403	3.22 at 403 K	^65^
[Tb_0.9900_Eu_0.0100_ (TCA)(phen)]·DEF	283–393	4.24 at 393 K	^66^
[Ln(ad)_0.5_(phth)(H_2_O)_2_] (Ln = 5Eu^3+^:10Tb^3+^)	303–423	1.21 at 303 K	^67^
[Eu_0.53_Tb_0.47_(Tfac)_8_]^2–^Na_2_^+^	273–373	2.70 at 353 K	^68^
[Tb_0.9_Eu_0.1_-L]	303–423	1.75 at 423 K	^69^
[Tb_0.08_Eu_0.92_-HPIDC-OX]	303–473	0.60 at 473 K	^70^
**[**Eu_0.19_Tb_0.81_PDDI]	313–473	0.37 at 473 K	^71^
[Tb_0.94_Eu_0.06_L_2_NO_3_(TPPO)_2_]	77–360	2.80 at 180 K	^72^
[Eu_0.2_Tb_0.8_(hbz)_3_(H_2_O)*_n_* (C_2_H_5_OH)*_m_*]	298–373	4.00 at 373 K	^73^
[Eu_0.05_Tb_1.95_-PDC]	293–333	1.37 at 333 K	^74^
[Eu_0.4_Tb_1.6_(keto)_6_(bpy)_2_]	283–343	4.72 at 343 K	this work

a Nomenclature: TCTZ = 2,4,6-tris­(4-carboxyphenyl)-1,3,5-triazine; H_3_TCA = 4,4′,4″-nitrilotribenzoic acid; bpy = 2,2′-bipyridyl; DEF= *N*,*N*-diethylformamide; phth^2–^ = phthalate; ad^2–^ = adipate; Htfac = 1,1,1-trifluoro-2,4-pentadione; H_2_L = 3-bis­(3-carboxyphenyl)­imidazolium; H_3_PIDC = 2-pyridin-4-yl-4,5-imidazoledicarboxylic acid; OX = sodium oxalate; H_4_PDDI = 5,5′-(pyridine-2,5-diyl)­diisophthalic acid; HL = 4,4,4-trifluoro-1-(furan-2-yl) butan-1,3-dione; TPPO = triphenylphosphine oxide; Hbz = 4-hydroxybenzoates; H_2_PDC = pyridine-3,5-dicarboxylic acid.

In this temperature interval, the maximum relative sensitivity (S_r_) exceeds 2.5% K^–1^ at 313.15 K. This S_r_ value surpasses those reported for many previously published systems (see Table 2), indicating that the mixed complex [Eu_0.4_Tb_1.6_(keto)_6_(bpy)_2_] is a highly promising candidate for luminescent thermometry in the physiological temperature range.

**Table 2:** Comparative Analysis of the Relative Sensitivity (*S*_r_) in the Physiological Range of Several Lanthanide-Based Ratiometric Materials^a^

complexes	range (K)	*S*_r_ (max) %K^–1^	references
[Eu_0.05_Tb_1.95_-PDC]	293–333	0.64 at 318 K	^74^
[Tb_0.99_Eu_0.01_(BDC)_1.5_(H_2_O)_2_]	300–320	0.37 at 318 K	^75^
[Tb_0.8_Eu_0.2_BPDA]	298–318	1.19 at 313 K	^76^
([C_2_mim][Tb(fod)_4_]_0.99985_.[C_2_mim][Eu(fod)_4_]_0.00015_)	309–318	4.10 at 314 K	^77^
[Eu_0.4_Tb_1.6_(keto)_6_(bpy)_2_]	308–318	2.52 at 313 K	this work

a Nomenclature: H_2_PDC = pyridine-3,5-dicarboxylic acid; BDC = 1–4-benzendicarboxylate; H_2_BPDA - biphenyl-3,5-dicarboxylate acid; C_2_mim = 1-methyl-3-ethylimidazolium; fod = tetrakis-6,6,7,7,8,8,8-heptafluoro-2,2-dimethyl-3,5-octanedionate.

### 3.4 Lifetimes Decay

The luminescence lifetimes of the mixed complex [Eu_0.4_Tb_1.6_(keto)_6_(bpy)_2_] (see Figure 9a,9b) were compared with those of the homobimetallic reference compounds [Eu_2_(keto)_6_(bpy)_2_] (Figure 10c) and [Tb_2_(keto)_6_(bpy)_2_] (Figure 10d). The decay curves were fitted with a monoexponential function, *I*(*t*) = *A* + *B*·exp­(−t/τ), where *I*(*t*) is the luminescence intensity at time, *A* and *B* are constants, t is the time and τ is the luminescence lifetime.^78^ For Tb^3+^ in [Tb_2_(keto)_6_(bpy)_2_] the lifetime is τ = 1.539 ± 0.003 ms, while in the mixed complex it decreases to τ = 1.211 ± 0.004 ms. On the other hand, Eu^3+^ in [Eu_2_(keto)_6_(bpy)_2_] exhibits τ = 1.236 ± 0.003 ms, which increases to τ = 1.571 ± 0.003 ms in the mixed complex. The reduction of the Tb^3+^ lifetime in the mixed complex suggests that part of the energy absorbed by Tb^3+^ may have been transferred to Eu^3+^. The concomitant increase in the Eu^3+^ lifetime in this system supports the possibility of Tb^3+^ → Eu^3+^ energy transfer.^79,80^ The lifetimes of the emitting states ^5^D_4_ (Tb^3+^) and ^5^D_0_ (Eu^3+^) were also measured at different temperatures (283–343 K), and the corresponding values are presented in Tables S2 and S3, respectively.

**Figure 9:**
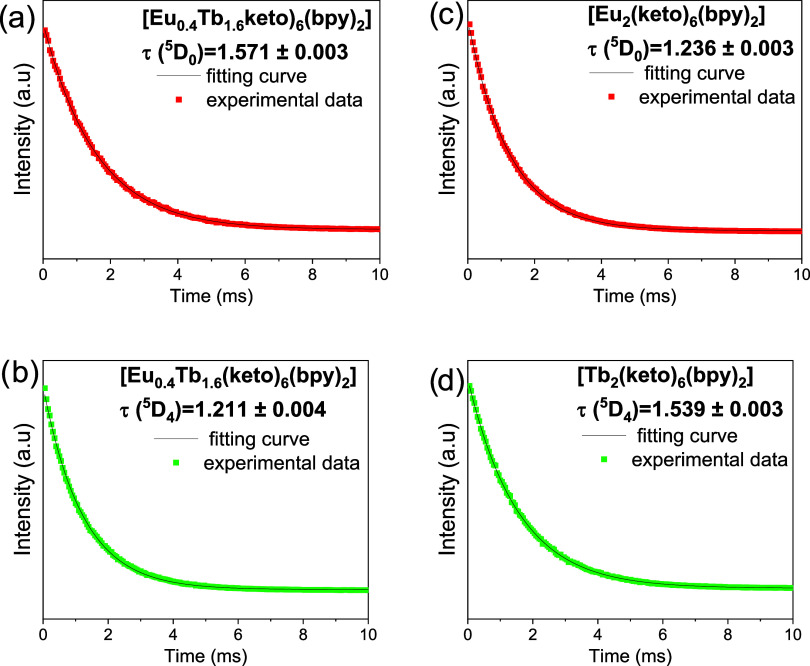
Luminescence decay curves of (a) Eu^3+^ ions in [Eu_0.4_Tb_1.6_(keto)_6_(bpy)_2_], (b) Tb^3+^ ions in [Eu_0.4_Tb_1.6_(keto)_6_(bpy)_2_], (c) [Eu_2_(keto)_6_(bpy)_2_], and (d) [Tb_2_(keto)_6_(bpy)_2_]. The data reveal an increase in the luminescence lifetime of Eu^3+^ ions in [Eu_0.4_Tb_1.6_(keto)_6_(bpy)_2_] compared to [Eu_2_(keto)_6_(bpy)_2_], whereas the lifetime of Tb^3+^ ions decreases in [Eu_0.4_Tb_1.6_(keto)_6_(bpy)_2_] relative to [Tb_2_(keto)_6_(bpy)_2_].

**Figure 10:**
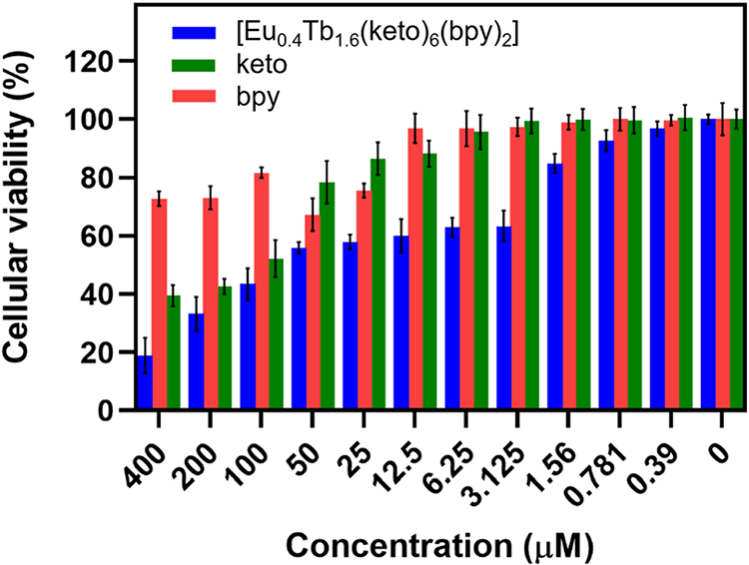
Cell viability of MRC-5 cells after 48 h of incubation with the mixed complex [Eu_0.4_Tb_1.6_(keto)_6_(bpy)_2_], keto (ketoprofen), and bpy (2,2′-bipyridine) at concentrations ranging from 0.39 to 400 μM, measured by the MTT assay.

### 3.5 Cellular Viability

The cytotoxicity of ketoprofen, 2,2′-bipyridine, and the complex [Eu_0.4_Tb_0.6_(keto)_6_(bpy)_2_], was evaluated in human lung fibroblasts (MRC-5) using the MTT assay, as shown in Figure 10. The IC_50_ values are reported in Table 3. MRC-5 fibroblasts represent a standard nontumoral model for preliminary biocompatibility evaluation of luminescent probes intended for biological imaging and intracellular sensing.

**Table 3:** IC_50_ Values (μM) for Mixed Complex and Their Corresponding Ligands in MRC-5 Cell Line

compound	IC_50_ (μM)
[Eu_0.4_Tb_1.6_(keto)_6_(bpy)_2_]	50.32 ± 0.52
keto	108 ± 0.65
bpy	>400

The complex [Eu_0.4_Tb_1.6_(keto)_6_(bpy)_2_] exhibited low cytotoxicity (IC_50_ = 50.32 μM), which places it at the border of commonly accepted safety thresholds for nontumoral cell lines used in bioimaging studies (values ≥ 50 μM are frequently considered acceptable for initial biocompatibility). Notably, many Eu^3+^/Tb^3+^ coordination complexes containing polypyridyl ligands report IC_50_ values in the 10–40 μM range for fibroblasts, meaning that the present system falls among the less cytotoxic members of this class of luminescent materials.^81,82^

The individual ligands further support this favorable profile: ketoprofen shows minimal cytotoxicity (IC_50_ = 108 μM), consistent with its established biological safety, while 2,2′-bipyridine exhibits no detectable toxicity within the tested range (IC_50_ > 400 μM). These results indicate that complexation does not amplify the intrinsic reactivity of the ligands, suggesting a chemically stable coordination environment that limits undesirable interactions with cellular components.

Overall, the cytotoxicity data demonstrate that the assembly of Eu^3+^/Tb^3+^ with ketoprofen and bpy yields a luminescent coordination complex with preserved cell viability, reinforcing its suitability for applications such as bioimaging and luminescent thermometry, where biocompatibility is a key requirement.

## 4 Conclusions

In this work, we synthesized a new mixed complex of Eu^3+^ and Tb^3+^ derived from the nonsteroidal anti-inflammatory drug ketoprofen and an *N*,*N*′-donor ligand. The complex was characterized by multiple analytical and spectroscopic techniques, which indicate isostructurality with the corresponding homobimetallic compounds. The ligands act as efficient sensitizers for both Eu^3+^ and Tb^3+^, and the photophysical behavior of the complex was investigated over a wide temperature range. The emission spectra display the characteristic transitions of Eu^3+^ and Tb^3+^, confirming successful incorporation of both ions into the mixed structure. A clear correlation between emission color and temperature was observed, demonstrating the material’s potential as a luminescent thermometer. The efficiency of Tb^3+^ → Eu^3+^ energy transfer increases with temperature, producing enhanced Eu^3+^ emission and a progressive red shift of the overall emission. This temperature-dependent behavior was analyzed using the ratio of the integrated areas of the ^5^D_4_ → ^7^F_5_ (Tb^3+^) and ^5^D_0_ → ^7^F_2_ (Eu^3+^) transitions under ligand-centered excitation. From these measurements, we calculated the thermometric parameters: the mixed complex [Eu_0.4_Tb_1.6_(keto)_6_(bpy)_2_] exhibited a maximum relative sensitivity of 4.72% K^–1^ at 343.15 K. Combined with excellent repeatability over ten consecutive heating–cooling cycles and low temperature uncertainty, these results highlight the complex as a promising high-precision solid-state luminescent temperature sensor. Finally, cell-viability assays in MRC-5 fibroblasts confirmed low cytotoxicity and good biocompatibility under the tested conditions, further supporting the potential of this material for biological and biomedical temperature-sensing applications.

## Supplementary Material



## Notes

Powder X-ray diffractograms, figures of excitation and emission spectra, thermometry experimental details, chromaticity coordinates, thermal uncertainty data, table of lifetime temperature-dependent (PDF)
