# Supplementary material for: A Biocompatible and High-Sensitivity Lanthanide Coordination Complex for Luminescent Temperature Sensor Constructed from Non-Steroidal Anti-Inflammatory Drug Ketoprofen
Source: ACS Omega. 2026 Feb 12;11(7):12105–16. doi: 10.1021/acsomega.5c11396 (PMC12946950; doi:10.1021/acsomega.5c11396)
Supplement: Supplementary file 1 [file ao5c11396_si_001.pdf]

A Biocompatible and High-Sensitivity Lanthanide Coordination Complex for Luminescent Temperature Sensor Constructed from Non-Steroidal Anti-inflammatory Drug Ketoprofen

Júlia Pereira de Oliveira Silva [a, b] *, *Molíria V. dos Santos* [c], *Roberta S. Pugina* [d], *Marina Paiva Abuçafy* [d], *Francisco R. Torres* [e], *José Maurício A. Caiut* [e] *and Lippy F. Marques* [a] *

[a] *Grupo de Química de Coordenação e Espectroscopia de Lantanídeos (GQCEL), Instituto de Química, Universidade do Estado do Rio de Janeiro, Rio de Janeiro-RJ, 20550-013, Brazil;*

[b] *Instituto Federal de Educação, Ciência e Tecnologia do Paraná (IFPR), Pitanga-PR, 85201-106, Brazil;*

[c] *BioSmart Nanotechnology, Faculdade de Engenharia Química, Universidade Estadual de Campinas – UNICAMP, Campinas, SP, 14808-162, Brazil;*

[d] *Instituto de Química, Universidade Estadual Júlio de Mesquita Filho – UNESP, Araraquara, SP, 14800-060, Brazil;*

[e] *Departamento de Química, Grupo de Nanomateriais e Sistemas Luminescentes, Faculdade de Filosofia, Ciências e Letras de Ribeirão Preto, Universidade de São Paulo (USP), 14040-901, Ribeirão Preto - SP, Brazil.*

* Corresponding author: Tel.: +55-21-23340563

E-mail address: lippymarquesuerj@gmail.com and juliahpereirah22@gmail.com

Keywords: Temperature Sensor; Lanthanide Complex; Luminescent Thermometer; High Sensitivity; Biocompatible.

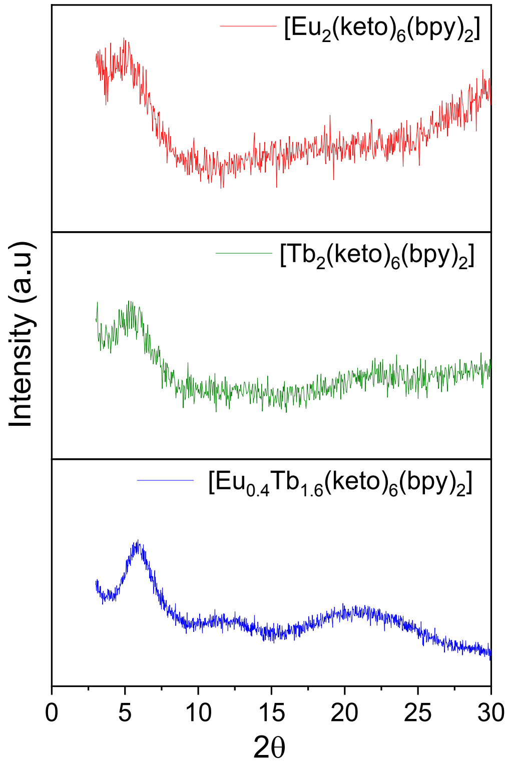

Figure S1. Powder X-ray diffractograms for the $[Eu_2(keto)_6(bpy)_2]$ [1], $[Tb_2(keto)_6(bpy)_2]$ [1] and, $[Eu_{0.4}Tb_{1.6}(keto)_6(bpy)_2]$ complexes.

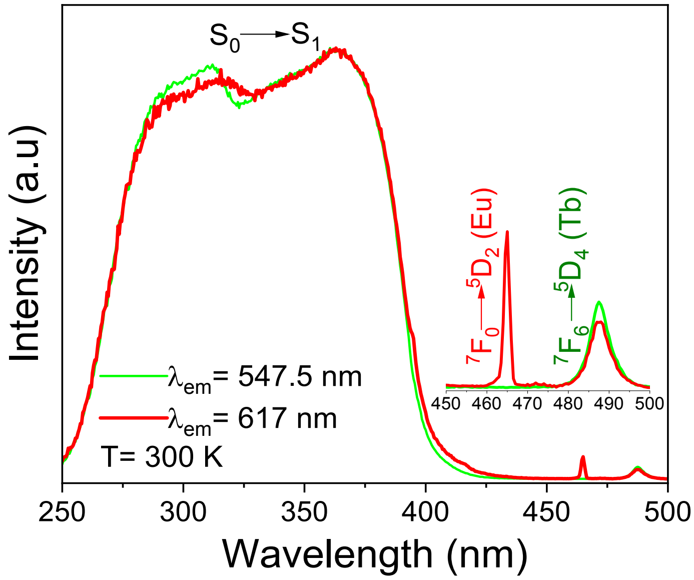

Figure S2. Excitation spectra of the $[Eu_{0.4}Tb_{1.6}(keto)_6(bpy)_2]$ complex in the solid state at room temperature (300 K), monitoring the Eu^{3+} $^5F_0 \rightarrow {}^5D_2$ emission (λ = 617.0 nm) and Tb^{3+} emission $^5F_6 \rightarrow {}^5D_4$ (λ = 547.5 nm).

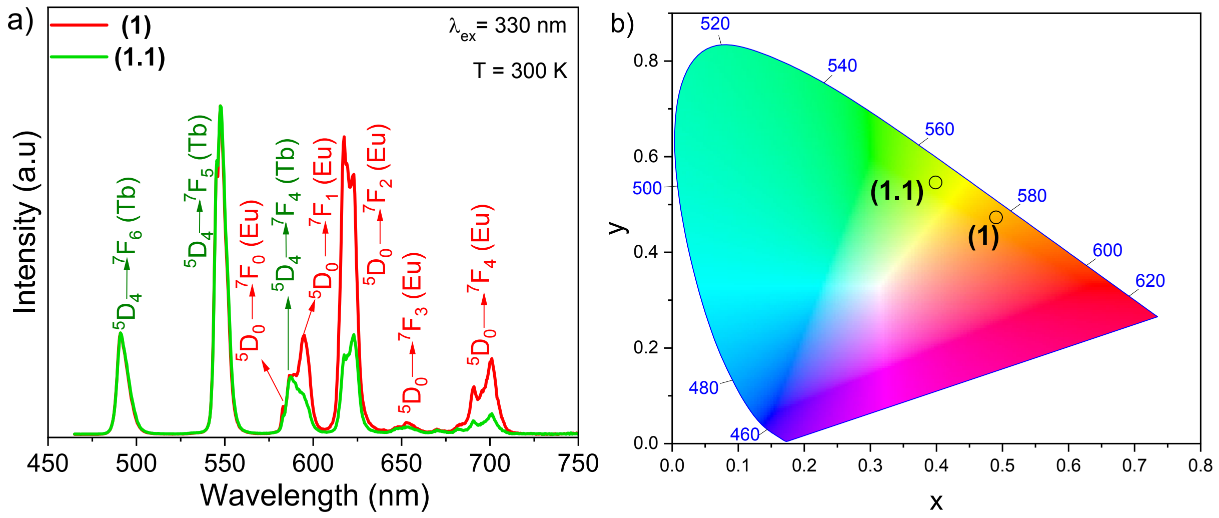

Figure S3. a) Emission spectra of the $[Eu_{0.4}Tb_{1.6}(keto)_6(bpy)_2]$ (1) (in red) and the physical mixture of $[Eu_2(keto)_6(bpy)_2]$ [1] + $[Tb_2(keto)_6(bpy)_2]$ [1] (1:1) (in green), showing the difference in the characteristic intensities of the Eu^{3+} ions and Tb^{3+} ions in the physical mixture and in the mixed complex. b) CIE chromaticity diagram showing the difference in the x,y emission color coordinates of (1) (x = 0.49026, y = 0.47318) and (1:1) (x = 0.39873, y = 0.54623).

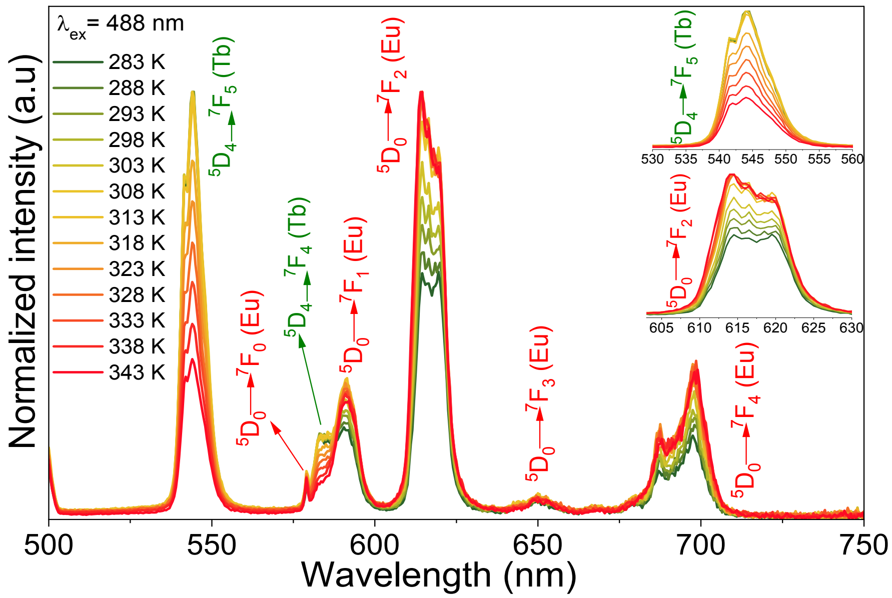

Figure S4. Emission spectra of the $[Eu_{0.4}Tb_{1.6}(keto)_6(bpy)_2]$ complex were recorded at various temperatures ranging from 283 to 343 K, under excitation at the Tb^{3+} (488 nm). The spectra were

measured in 5 K increments. The inset highlights the temperature-dependent variations in the Tb^{3+} ($^5D_4 \rightarrow {}^7F_5$) and Eu^{3+} ($^5D_0 \rightarrow {}^7F_2$) electronic transition intensities.

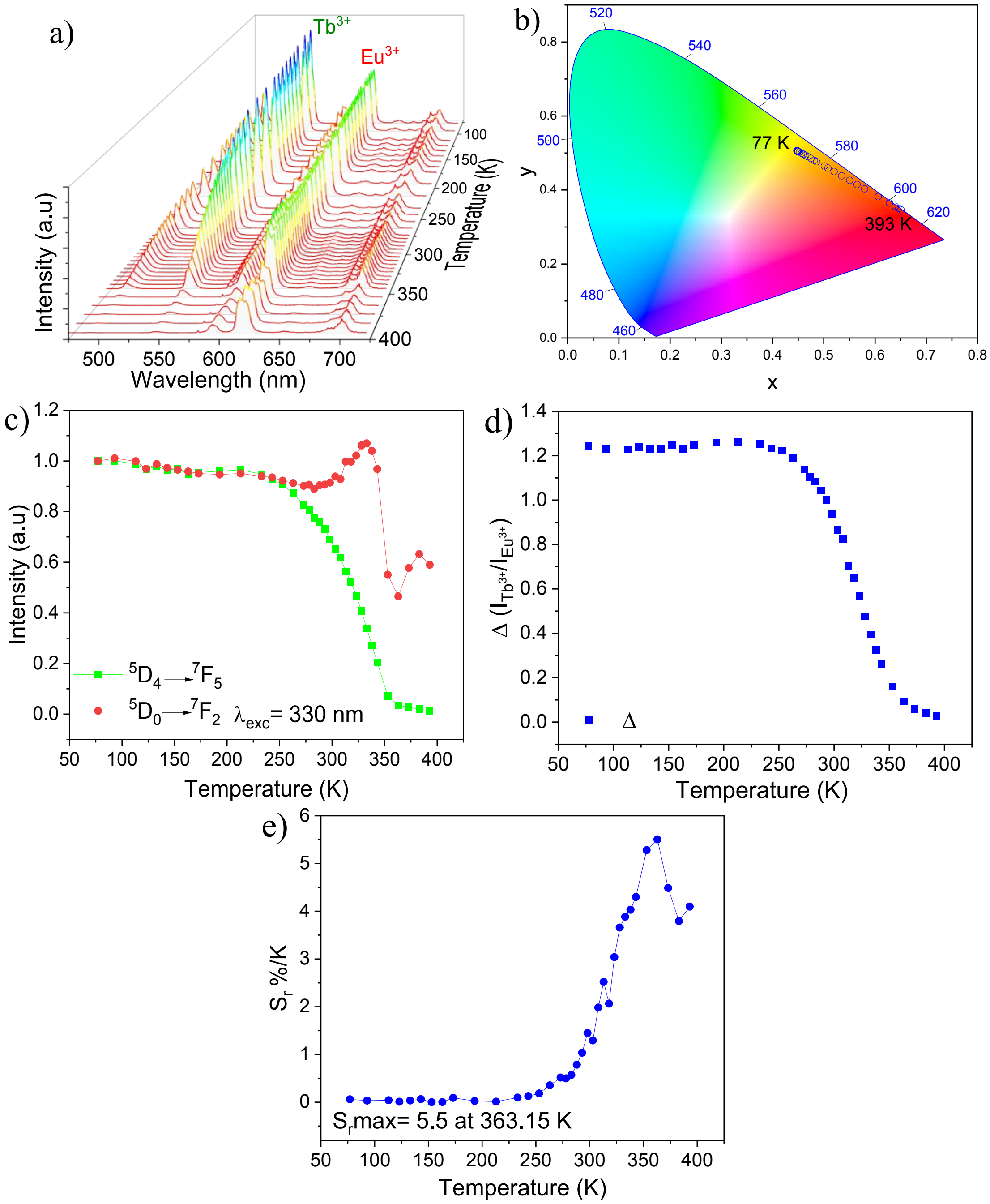

Figure S5. a) Emission spectra of the [$Eu_{0.4}Tb_{1.6}$(keto)$_6$(bpy)$_2$] complex recorded between 283 and 343 K under excitation at the ligand absorption band (330 nm). Spectral measurements were performed over a broader temperature range (77–393 K), with 10 K increments from 93–273 K

and 343– 393 K, and 5 K increments from 273–343 K. b) CIE 1931 chromaticity diagram illustrating the temperature-induced color evolution from greenish-yellow (77 K) to red (393 K). c) Normalized intensities of the $^5D_4 \longrightarrow {}^7F_5$ and $^5D_0 \longrightarrow {}^7F_2$ transitions in the 283–343 K range. d) Temperature-dependent intensity ratio of Tb^{3+} (547.7 nm) to Eu^{3+} (617 nm).

Table S1. Chromaticity coordinates of the $[Eu_{0.4}Tb_{1.6}(keto)_6(bpy)_2]$ complex.

**Temperature (K)**	**Coordinates**	
	**x**	**y**
77	0.44817	0.50480
93	0.44897	0.50425
113	0.44913	0.50430
123	0.44855	0.50488
133	0.44887	0.50459
143	0.44915	0.50465
153	0.44795	0.50550
163	0.44921	0.50449
173	0.44818	0.50541
193	0.44730	0.50620
213	0.44740	0.50642
233	0.44791	0.50635
243	0.44916	0.50524
253	0.45012	0.50453
263	0.45278	0.50252
273	0.45653	0.49950
278	0.45929	0.49736
283	0.46130	0.49589
288	0.46449	0.49333
293	0.46836	0.49021
298	0.47482	0.48570
303	0.48167	0.48005
308	0.48597	0.47663
313	0.50122	0.46495
318	0.50792	0.45931
323	0.52006	0.44988
328	0.53476	0.43842
333	0.55041	0.42604
338	0.56487	0.41476
343	0.57944	0.40337
353	0.60633	0.38206

363	0.62809	0.36481
373	0.63944	0.35571
383	0.64633	0.35039
393	0.65077	0.34680

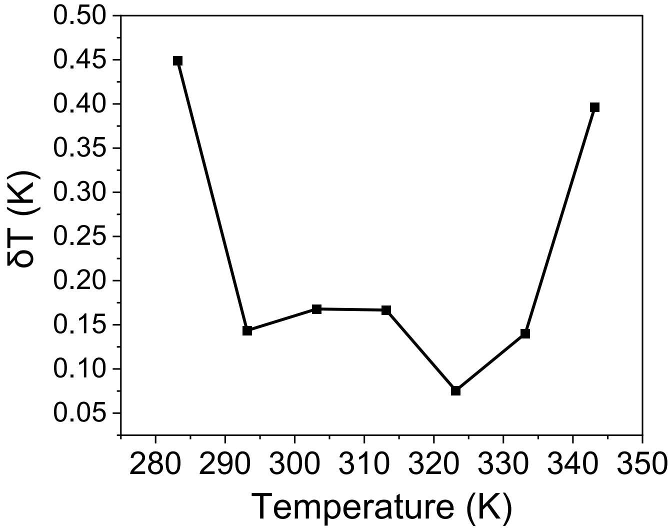

Figure S6. Evaluating Thermal Uncertainty (δT) of the [$Eu_{0.4}Tb_{1.6}(keto)_6(bpy)_2$].

Table S2. Lifetime monitored at the $^5D_4 \rightarrow {}^7F_5$ (λ = 547.5 nm) transition under excitation at the ligand absorption band (330 nm), showing temperature-dependent lifetime variations from 283 to 343 K.

Temperature (K)	$\tau(^5D_4)$ (ms)
283	1.278
288	1.251
293	1.221
298	1.185
303	1.154
308	1.105
313	1.069
318	1.027
323	0.982

328	0.933
333	0.881
338	0.838
343	0.789

Table S3. Lifetime monitored at the $^5D_0 \rightarrow {}^7F_2$ transition (λ = 617 nm) under excitation at the ligand absorption band (330 nm), showing temperature-dependent variations between 283 and 343 K.

Temperature (K)	$\tau(^5D_0)$ (ms)
283	1.534
288	1.560
293	1.570
298	1.600
303	1.610
308	1.628
313	1.623
318	1.642
323	1.688
328	1.675
333	1.653
338	1.677
343	1.669

Reference

[1] N. A. Guilherme *et al.*, "First Eu^{3+}, Gd^{3+} and Tb^{3+} complexes containing the non-steroidal anti-inflammatory drug ketoprofen and N,N-donors ligands: Synthesis, solid state

characterization and photoluminescence studies," *J Lumin*, vol. 269, May 2024, doi: 10.1016/j.jlumin.2024.120472.
